# Clinical, Anthropometric and Biochemical Characteristics of Patients with or without Genetically Confirmed Familial Hypercholesterolemia

**DOI:** 10.5935/abc.20180005

**Published:** 2018-02

**Authors:** Andrea De Lorenzo, Juliana Duarte Lopes da Silva, Cinthia E. James, Alexandre C. Pereira, Annie Seixas Bello Moreira

**Affiliations:** 1 Instituto Nacional de Cardiologia, Rio de Janeiro, RJ - Brazil; (2) Laboratório de Genética e Cardiologia Molecular, Instituto do Coração (InCor) - Faculdade de Medicina da Universidade de São Paulo, São Paulo, SP - Brazil

**Keywords:** Hyperlipoproteinemia Type II, Body Weights and Measurements, LDL Lipoproteins, Dyslipidemias, Mutation, Phenotype

## Abstract

**Background:**

Familial hypercholesterolemia (FH) is a common autosomal dominant disorder, characterized by a high level of low-density lipoprotein cholesterol (LDL-C) and a high risk of premature cardiovascular disease.

**Objective:**

To evaluate clinical and anthropometric characteristics of patients with the familiar hypercholesterolemia (FH) phenotype, with or without genetic confirmation of FH.

**Methods:**

Forty-five patients with LDL-C > 190 mg/dl were genotyped for six FH-related genes: LDLR, APOB, PCSK9, LDLRAP1, LIPA and APOE. Patients who tested positive for any of these mutations were considered to have genetically confirmed FH. The FH phenotype was classified according to the Dutch Lipid Clinic Network criteria.

**Results:**

Comparing patients with genetically confirmed FH to those without it, the former had a higher clinical score for FH, more often had xanthelasma and had higher LDL-C and apo B levels. There were significant correlations between LDL-C and the clinical point score for FH (R = 0.382, p = 0.037) and between LDL-C and body fat (R = 0.461, p = 0.01). However, patients with mutations did not have any correlation between LDL-C and other variables, while for those without a mutation, there was a correlation between LDL-C and the clinical point score.

**Conclusions:**

LDL-C correlated with the clinical point score and with body fat, both in the overall patient population and in patients without the genetic confirmation of FH. In those with genetically confirmed FH, there were no correlations between LDL-C and other clinical or biochemical variables in patients.

## Introduction

Familial hypercholesterolemia (FH) is characterized by a high level of low-density lipoprotein cholesterol (LDL-C) and a high risk of premature cardiovascular disease.^1^ It is a common autosomal dominant disorder, affecting up to 1 in 200-250 people in its heterozygous form.^2^ According to the Dutch Lipid Clinic Network, the clinical diagnosis of FH (FH phenotype) is based on high LDL-C and a score in which points are assigned for family history of hyperlipidemia or heart disease, clinical characteristics such as tendinous xanthomata, elevated LDL cholesterol, and/or an identified mutation. A total point score greater than eight is considered "definite" FH, 6-8 is "probable" FH, and 3-5 is "possible" FH.^3^

Despite being helpful as they provide a standardization of the diagnosis of the FH phenotype, scores may not necessarily result in consistent diagnoses of FH, as cholesterol levels for FH patients overlap with those of the general population. Genetic diagnosis is considered evidence of definite FH according to some criteria.^1^ Mutations in 3 genes- the LDL-receptor gene (LDLR), the gene coding for apolipoprotein B and the gene encoding the proprotein convertase subtilisin/kexin type 9-are usually responsible for FH.^4-6^ However, other mutations have been identified in the LDLR gene, as well as mutations in other genes leading to the clinical FH phenotype, and there is also evidence that some mutations lead to more severe manifestations of FH than others. Additionally, a large proportion of the patients with a clinical diagnosis of FH do not have a detectable mutation in any of these genes.^7,8^

In view of the complexity of this scenario, there is continuing need for additional information on the clinical and laboratory profile of patients with either genetically defined FH or with only the phenotype of FH, since such data might help optimize patient management, in the sense of their cardiovascular risk burden. Therefore, this study sought to evaluate clinical and anthropometric characteristics of patients with or without genetic confirmation of FH.

## Methods

### Study population

This was a cross-sectional study of adult outpatients with severe hypercholesterolemia recruited at the National Institute of Cardiology in Rio de Janeiro, Brazil. Subjects with LDL-C > 190 mg/dl and in use of lipid-lowering drug were selected after review of the lipid panel results over 6 months. These patients were invited by phone call to take part in the study, and those with acute coronary syndromes or myocardial revascularization in the previous 30 days, autoimmune diseases, thyroid disorders, chronic renal failure, liver diseases, malignancy, using steroids, or pregnant or breastfeeding were excluded. For this study, a convenience sample was used, including all patients who had been genetically screened to date. Once considered eligible, all participants read and signed an informed consent document approved by the institutional Ethics Committee. The study was undertaken in accordance with the Helsinki Declaration of 1975, revised in 2000.

The study patients underwent clinical evaluation and peripheral blood collection. The FH phenotype was classified according to the Dutch Lipid Clinic Network criteria.^3^ Prior cardiovascular disease was defined as a history of myocardial infarction, evidence of obstructive coronary artery disease at coronary angiography (> 50% stenosis of any epicardial coronary artery), myocardial revascularization (either percutaneous or coronary artery bypass surgery) or stroke. Hypertension was defined as blood pressure ≥ 140/90 mmHg and/or antihypertensive drug use. Diabetes mellitus was defined by history and use of insulin or oral hypoglycemic medications, or fasting glucose levels > 126 mg/dl.

### Anthropometric measurement

All patients underwent assessment of body composition. Body mass index (BMI) was calculated as weight in Kg/ (height)^2^. Body composition (body fat percentage [%], visceral fat area [cm^2^] and phase angle [degrees]) was estimated by bioelectrical impedance, using the multifrequency analyzer octopolar (In-Body 720; Biospace). The measurements were made with the patient in the supine position, with the arms lying parallel and separated from the trunk and with the legs separated, so that the thighs were not touching. Two electrodes were placed on the hand and wrist and another two were positioned on the foot and ankle of the non-dominant side of the body. An electrical current measured at six different frequencies (1, 5, 50, 250, 500 and 1000 KHz) was introduced into the subject, and resistance and reactance were measured. The phase angle was calculated according to the following equation: Phase Angle = arctangent (inductance / resistance) × 180º/ π.^9^

### Laboratory measurements

For biochemical testing, venous blood samples were obtained in the morning after 12 h of fasting. The patients took their usual medications on the morning of the tests. Laboratory evaluations were performed by an automated method (ARCHITECT ci8200, Abbott ARCHITECT®, Abbott Park, IL, USA) using commercial kits (Abbott ARCHITECT c8000®, Abbott Park, IL, USA). Serum triglyceride levels, total cholesterol, LDL cholesterol (LDL-C), HDL-cholesterol (HDL-C), apolipoproteins A (apo A) and B (apo B) and C-reactive protein (CRP) were evaluated.

Genomic DNA was extracted from peripheral blood following a standard salting-out procedure. All DNA stock samples were quantified with Qubit dsDNA BR Assay Kit (Thermo Fisher) and diluted to 10 ng/ul for enrichment with Ion AmpliSeq Custom Kit (Thermo Fisher). Samples were enriched for six FH-related genes: LDLR, APOB, PCSK9, LDLRAP1, LIPA and APOE.

Patients who tested positive for any of these mutations were considered to have genetically confirmed FH. Target regions were considered as coding exons plus 10bp of introns up and downstream. Templates were prepared on Ion One Touch System and sequenced in Ion Torrent PGM ® platform, with 32 samples per run in a 316v2 Ion Chip. All FASTQ files were imported to CLC Genomics Workbench 9.5 (QIAGEN) and analyzed in a custom pipeline.

Minimum quality requirements for variant call were: Base quality of PhredQ ≥ 20; Target-region coverage ≥ 10x; Frequency of variant allele ≥ 20% and bidirectional presence of variant allele. After polymorphism filtering with control populations (NHLBI-ESP6500, ExAC and 1000Genomes), all potential mutations were consulted for previous description in ClinVar, Human Genome Mutation Database (HGMD), British Heart Foundation and Jojo Genetics databases. Functional impact prediction was performed with SIFT, PROVEAN and PolyPhen-2 and mutations without previous description should be pointed as damaging in at least two algorithms to be considered as potentially pathogenic. Individuals with negative results were also screened for large insertions and deletions via MLPA (MRC-Holland).

### Statistical analysis

Continuous data were analyzed using two-tailed unpaired Student's t test or Mann-Whitney's test, and categorical variables with *chi*-squared test. *Kolmogorov* - *Smirnov test was performed t* o determine whether sample data was normally distributed. Continuous variables are reported as means ± standard deviations, and categorical variables are presented as percentages. Correlations between continuous variables were analyzed with Pearson's test. Analyses were performed with SPSS software, version 21.0, and p values < 0.05 were considered statistically significant. Statistical review of the study was performed by a biomedical statistician.

## Results

Forty-five patients with LDL-C > 190 mg/dl were studied, of which fifteen had positive testing for familial hypercholesterolemia and thirty had negative. Comparing patients with genetically confirmed FH to those without it (Table 1), the former had a higher clinical score for FH, were more frequently considered to have definite FH, and more often had xanthelasma. Of note, the prevalence of prior coronary artery disease or stroke were not significantly different between patients with or without the genetic diagnosis of FH. Mean LDL-C and apo B levels were higher in patients with a molecular diagnosis of FH (Table 2).

**Table 1:** Demographic, anthropometric and clinical characteristics of patients with positive or negative genetic testing for familial hypercholesterolemia

	Positive (n = 15)	Negative (n = 30)	p-value
Age (years)	51.7 ± 14.4	55.6 ± 12.6	0.376
Weight	71.4 ± 16.1	70.8 ± 15.7	0.906
Body mass index (Kg/m²)	27.9 ± 6.1	28.3 ± 5.1	0.784
Body fat (%)	39.1 ± 9.4	35.6 ± 8.2	0.262
Visceral fat area (cm²)	110.3 ± 34.0	104.6 ± 34.3	0.639
Waist circumference (cm)	95.6 ± 10.6	96.5 ± 11.9	0.589
Hip circumference (cm)	104.4 ± 11.6	102.8 ± 12.1	0.676
Women	14 (93.3)	18 (60.0)	0.019*
Clinically defined FH	10 (66.7)	4 (13.3)	0.001*
Score	9.64 ± 2.16	4.35 ± 1.58	0.001*
**Risk factors and clinical data**			
Hypertension	8 (53.3)	20 (71.4)	0.197
Diabetes	3 (20.0)	6 (21.4)	0.619
Obesity	7 (46.7)	9 (30.0)	0.219
Smoking	0	3 (10.7)	0.265
Corneal arch	3 (20.0)	1 (3.7)	0.122
Xanthomata	0	0	
Xanthelasma	3 (20.0)	0 (0)	0.04*
Angina	6 (40.0)	12 (42.0)	0.559
History of stroke	0 (0)	1 (3.6)	0.651
History of myocardial infarction	3 (20.0)	11 (39.3)	0.173
Prior coronary angioplasty	3 (20.0)	11 (40.7)	0.153
Prior coronary bypass	4 (26.7)	5 (17.9)	0.381

Numbers are n (%), for categorical variables, or mean ± SD, for continuous variables;

(*) p < 0.05; FH: familial hypercholesterolemia.

**Table 2:** Laboratory data of patients with positive or negative genetic testing for familial hypercholesterolemia

	Positive (n = 15)	Negativo (n = 30)	p-value
Total cholesterol (mg/dL)	263.1 ± 93.1	231.0 ± 57.4	0.417
LDL-C (mg/dL)	208.1 ± 41.8	151.4 ± 50.6	0.002*
HDL-C (mg/dl)	52.2 ± 9.7	50.1 ± 12.0	0.617
Apo A1 (mg/dL)	139.3 ± 19.9	140.1 ± 22.9	0.916
Apo B (mg/dL)	138.7 ± 30,2	106.3 ± 31.6	0.005*
Triglyceride (mg/dL)	127.9 ± 52.1	144.6 ± 73.5	0.484
CRP (mg/dL)	0.4 ± 0.7	0.3 ± 0.6	0.707
Glycemia (mg/dL)	116.4 ± 79.9	107.5 ± 48,2	0.667

Numbers are n (%), for categorical variables, or mean ± SD, for continuous variables;

(*) p < 0.05; Apo A1: apolipoprotein A1; Apo B: apolipoprotein B; CRP-C: reactive protein; HDL-C: high-density lipoprotein cholesterol; LDL-C: low-density lipoprotein cholesterol.

When the correlations between LDL-C levels and other clinical, demographic and anthropometric variables were examined, there was a weak, although significant correlation between LDL-C and the clinical point score (R = 0.382, p = 0.037) and a moderate and significant correlation between LDL-C and body fat (R = 0.461, p = 0.01). However, when patients were stratified according to genetic testing positivity, those with any of the studied mutations did not show any correlation between LDL-C and other variables, while for those without a mutation, there was a moderate, statistically significant correlation between LDL-C and the clinical point score (R = 0.554, p = 0.01), as well as a borderline significant, moderate correlation between LDL-C and body fat (R = 0.441, p = 0.05).

## Discussion

FH is a disorder of cholesterol metabolism and indeed one of the most common inherited disorders.^2,10^ Rates of premature cardiovascular disease are much higher in patients with FH, but long-term drug therapy has the potential to lower cardiovascular event rates in patients with FH, leading to similar rates to those found in the general population.^11^ Since effective primary prevention in the setting of FH requires its early diagnosis, the largest knowledge we have on this disease, the best we may recognize it and accomplish adequate patient management.

In this study, patients with genetically confirmed FH had, as expected, a higher clinical score for FH. In addition, they had more clinical evidence of severe hypercholesterolemia such as xanthelasma, possibly since the monogenic group have had severely elevated LDL-C level since birth, and thus, a greater cumulative "LDL-C burden".^12^ Finally, LDL-C and Apo B levels were higher than in those patients with negative genetic testing, as previously demonstrated.^13,14^ Apo B is the main protein constituent of lipoproteins such as VLDL and LDL, and concentrations of Apo B tend to mirror those of LDL-C.^15^ Plasma levels of apolipoprotein B represent all atherogenic lipoproteins in the circulation; however, because every atherogenic particle contains a single apolipoprotein B molecule, Apo B levels also provide an accurate reflection of the number of atherogenic particles.^16^

Of note, LDL-C levels were correlated with the clinical point score and with body fat, both in the overall patient population and in patients without the genetic confirmation of FH. In those with genetically confirmed FH, there were no correlations between LDL-C and other clinical or biochemical variables in patients. This might suggest that the former might have less severe forms of FH related to other mutations, or severe hypercholesterolemia due to other etiologies, and in those cases the level of LDL-C would be also associated with modifiable or environmental factors. In contrast, in patients with FH, the severity of the derangements caused by the mutations would be the predominant factor determining LDL-C levels, what would turn other correlations with anthropometric or biochemical variables less significant.

This study is limited by the small sample size, which turns the results hypothesis-generating. Importantly, it may be possible that a proportion of the patients have a mutation in whomever as a yet unidentified gene. With standard molecular diagnostic techniques, a known mutation can be detected in 20-30% of patients with possible FH and 60-80% of patients with definite FH.^17,18^ Since approximately 2/3 of patients have possible FH, no mutations are detected in about 60% of tested patients with this disorder^17^ what has led to a search for additional FH-causing genes. However, some clinically diagnosed cases of FH may be polygenic, due to the inheritance of a greater than average number of common LDL-C raising alleles (each causing a slight effect) leading to an increase in LDL-C above the diagnostic cut off.^19^

## Conclusions

The present results suggest that in patients with severe hypercholesterolemia and the FH phenotype, even in the absence of genetic confirmation of FH, patient management should have special attention directed towards modifiable factors associated with LDL-C, as body fat. A decrease in body fat might determine a reduction of LDL-C, what is known to decrease cardiovascular risk.^20^
